# αß T-cell depleted haploidentical stem cell transplantation for pediatric and young adult patients with transfusion-dependent thalassemia

**DOI:** 10.1038/s41409-025-02546-w

**Published:** 2025-03-18

**Authors:** Katharina Kleinschmidt, Gina Penkivech, Anja Troeger, Juergen Foell, Tarek Hanafee-Alali, Stefanie Leszczak, Marcus Jakob, Sonja Kramer, Silke Kietz, Petra Hoffmann, Claudia Behrendt-Böhm, Carina Kaess, Andreas Brosig, Robert Offner, Daniel Wolff, Selim Corbacioglu

**Affiliations:** 1 Department of Pediatric Hematology, Oncology and Stem Cell Transplantation, University of Regensburg, Franz-Josef-Strauss Allee 11, 93053 Regensburg, Germany; 2 Department of Internal Medicine III, University of Regensburg, Regensburg, Germany; 3 Leibniz Institute for Immunotherapy (LIT), Regensburg, Germany; 4 Institute of Clinical Chemistry and Laboratory Medicine, University of Regensburg, Regensburg, Germany

**Keywords:** Translational research, Anaemia

## Abstract

Life expectancy of patients with severe transfusion-dependent beta-thalassemia (TDT) remains below that of the general population. Allogenic hematopoietic stem cell transplantation (HSCT) is the standard curative treatment. Due to the paucity of matched donor (MD) availability, haploidentical HSCT (haplo-HSCT) is a reasonable alternative. Twenty patients with TDT (median age 10 years; range 2–23) received either a matched sibling donor (MSD; *n* = 7) or a haplo-HSCT (*n* = 13) in a single center (Regensburg, Germany) between 2016 and 2022, including two patients referred for a haplo-HSCT as rescue failing prior MD- and haplo-HSCT, respectively. The conditioning regimen consisted of anti-thymocyte globulin (ATG; Grafalon®), treosulfan, thiotepa, and fludarabine (FTT). Immunosuppression consisted of a calcineurin inhibitor and mycophenolate mofetil (MMF). At a median follow-up of 37 months (range 6–90), overall survival (OS) was 100% with a disease-free survival (DFS) of 100% in MSD and 92% in haplo-HSCT, respectively. Two patients in haplo-HSCT experienced graft failure, one achieving DFS after a second haplo-HSCT. No acute graft-versus-host disease (aGvHD) ≥ °III or severe chronic GvHD (cGvHD) were observed. No sinusoidal obstruction syndrome (SOS) was observed in this high-risk population. Treosulfan-based T-cell depleted haplo-HSCT can achieve comparable OS and DFS even in young adult TDT patients with no SOS/VOD.

## Introduction

Beta-thalassemia is caused by over 350 mutations resulting in reduced or absent β-globin synthesis leading to an ineffective erythropoiesis and transfusion-dependency [1, 2]. Subsequently iron overload and systemic organ damage develop despite optimal supportive care [1, 2] compromising quality of life and life expectancy compared to that of the general population [3]. Consecutively, transfusion-dependent ß-thalassemia (TDT) has an absolute indication for a curative approach such as an allogenic hematopoietic stem cell transplantation (HSCT).

HSCT from a matched donor (MD) represents the current standard of care for TDT [4, 5]. In addition, HSCT is proving to be a cost-effective treatment option, as lifelong transfusion dependency and optimal supportive care are beyond the healthcare resources of most non-industrialized countries [5]. In children undergoing MD HSCT, an overall survival (OS) and event-free survival (EFS) of >90% and >82%, respectively was recently reported in a large series [6]. However, HSCT should be performed before iron overload and related complications develop, optimally in patients below the age of 14 [4] or even < 6 years of age [7]. OS and EFS for adults and patients assigned to a higher Pesaro classification remain poor, with 2-year OS in adults of 84.4% [8]. MSD availability is limited so that the probability of finding a suitable donor can be below 25% [9, 10] and below 10% in ethnic minorities [11]. Consequently, alternative donor HSCT regimens are urgently needed. A hyperplastic bone marrow, frequent alloimmunization due to chronic transfusions and anti-HLA antibodies increase the risk of primary or secondary graft failure (GF) [12, 13]. Iron overload with significant organ damage, particularly to the heart and liver [14] lead to high incidences of sinusoidal obstruction syndrome/veno-occlusive disease (SOS/VOD), exacerbated by a busulfan-based conditioning regimen [15].

Haploidentical HSCT (haplo-HSCT) offers a readily available curative treatment option for almost all patients. Currently, two different haplo-HSCT regimens are in use: ex vivo T-cell depletion via TCRαβ/CD19^+^ depletion (formerly CD3^+^/CD19^+^) (T-haplo-HSCT) [12, 16, 17]; and in vivo T-cell depletion with post-transplantation cyclophosphamide (post-Cy) [12, 18].

While the post-Cy concept has reported outcome rates of about 95% OS and DFS; it is associated with high incidences of acute and chronic GvHD (aGvHD: 31–42%, cGvHD: 17–40%), particularly detrimental in patients with non-malignant diseases [19, 20]. Few data are available for T-haplo-HSCT in patients with TDT. Two studies report OS and DFS rates ranging from 84–90% and 61–69%, respectively [13, 21] utilizing a busulfan-based conditioning regimen. In recent years, treosulfan, an alkylator with excellent immunosuppressive and myeloablative capacity and a low toxicity profile is an emerging standard for the conditioning in non-malignant and malignant diseases [22–24]. Treosulfan also demonstrated to be safe and highly effective in the haploidentical setting for sickle cell disease (SCD) [24], currently being evaluated in a prospective trial (EudraCT No. 2018-002652-33).

Here, we report the outcome of 20 TDT patients receiving a T-depleted haplo-HSCT (mostly TCRαβ/CD19^+^) compared to a MSD HSCT to determine the feasibility, safety, and efficacy of an in vitro T-depleted haplo-HSCT in TDT.

## Patients

Twenty patients with TDT (median age 10 years; range 2-23 years) were transplanted between 2016 and 2022 in a single center (Regensburg, Germany). Thirteen patients with no available donor according to high-resolution molecular typing (10/10) received 14 T-haplo-HSCT (CD3^+^/CD19^+^: *n* = 3 and TCRαβ/CD19^+^: *n* = 11) compared to seven patients with a MSD HSCT.

Patients’ characteristics are summarized in Table 1. Iron overload was defined by magnetic resonance imaging (MRI), ultrasound and/or liver biopsy. Portal fibrosis and hepatomegaly >2 cm were diagnosed via ultrasound. Liver biopsies with histological classification according to Ishak et al. [25] were performed in all cases with a hepatic iron overload and signs of fibrosis. Six patients were categorized as Pesaro class I, nine to class II, and three to class III. All patients were transfusion-dependent (every 3–4 weeks) prior to HSCT. Patients with substantial iron overload received an intensified chelation with two chelators for ‘downstaging’ over several weeks prior to HSCT.

**Table 1:** Characteristics of patients and donors undergoing T-depleted haplo-HSCT and MSD HSCT.

Pat.	Age (years)	Sex	ß-Thal subtype	Thal-associated complications/ secondary diagnosis	MRI Iron (µmol/g)	Pesaro class	Ishak	Donor age (years)	Donor sex	Donor relation	HLA match	T-cell depletion	BG compatibility
**Haplo-HSCT**													
1^b^	23	F	β^0^/β^0^	ED, CD, HSM, S	158	II	n.a.	43	F	Mother	5/10	CD3/CD19	Compatible
2a^a^	10	M	β^0^/β^0^	CD	35	I	n.a.	50	M	Father	5/10	CD3/CD19	Major incomp.
2b^a^	11	M	β^0^/β^0^	CD	35	I	n.a.	39	F	Mother	5/10	CD3/CD19	Minor incomp.
3^b^	8	M	Β^+^/β^+^	–	99	II	n.a.	28	F	Mother	6/10	TCRαβ/CD19	Compatible
4^b^	3	M	β^0^/β^+^	CD, GF	114	II	0	40	M	Father	5/10	TCRαβ/CD19	Major incomp.
5	21	M	β^0^/β^0^	ED, H, S, IT	n.a.	II	5	41	F	Mother	5/10	TCRαβ/CD19	Compatible
6	6	F	β^0^/β^0^	N	n.a.	I	n.a.	41	F	Mother	5/10	TCRαβ/CD19	Major incomp.
7	2	F	β^0^/β^+^	–	n.a.	I	n.a.	36	M	Father	6/10	TCRαβ/CD19	Compatible
8	11	F	β^0^/β^0^	ED, HSM	n.a.	I	n.a.	37	M	Father	7/10	TCRαβ/CD19	Major incomp.
9^b^	6	M	β^0^/β^0^	ED, GF, H	268	II	1	37	F	Mother	5/10	TCRαβ/CD19	Minor incomp.
10	11	M	β^0^/β^0^	ED	13	II	n.a.	38	M	Father	7/10	TCRαβ/CD19	Compatible
11	11	M	β^0^/β^0^	Allo-I, HSM	179	II	n.a.	35	F	Mother	7/10	TCRαβ/CD19	Minor incomp.
12^b^	8	M	β^0^/β^0^	–	64	II	n.a.	36	F	Mother	6/10	TCRαβ/CD19	Compatible
13	13	M	β^0^/β^0^	S	84	II	n.a.	36	F	Mother	5/10	TCRαβ/CD19	Minor incomp.
**MSD-HSCT**													
1	4	M	β+/β°	HSM	n.a.	I	n.a.	15	M	Brother	10/10	–	Minor incomp.
2^b^	11	M	β°/β°	ED	n.a.	III	n.a.	21	F	Sister	10/10	–	Compatible
3	7	M	β+/β°	–	n.a.	I	n.a.	12	M	Brother	10/10	–	Compatible
4^b^	10	F	β+/β°	ED, H, HSM	429	III	2	16	M	Brother	10/10	–	Minor incomp.
5	12	M	β°/β°	ED, IT, N	240	II	0	13	M	Brother	10/10	–	Compatible
6^b^	12	F	β+/β°	H, HSM, N	571	III	2	25	M	Brother	10/10	–	Compatible
7^b^	10	F	β°/β°	ED, HSM	140	II	n.a.	14	M	Brother	10/10	–	Compatible

*Allo-I* allo-immunization, *β° ß*^*+*^ ß-thalassemia genotype variants, *CD* cardiac dysfunction (including cardiomegaly and impaired cardiac pump function, pulmonary stenosis), *ED* endocrine dysfunction (including diabetes mellitus type 1, hypoparathyroidism, hypothyroidism, adrenal insufficiency, growth retardation, hypogonadism, pubertas tarda), *F* female, *GF* graft failure after 1^st^ SCT, *H* hepatopathy (including liver fibrosis/ cirrhosis and/or functional impairment*)*, *HLA* human leucocyte antigens, *HSM* hepato- and/or splenomegaly, *IT* immune thrombocytopenia, *M* male, *N* nephropathy, *n.a*. non applicable, *S* splenectomy.

^a^Same patient

^b^Patients received intensified iron chelation therapy with deferasirox (Exjade®) and deferiprone (Ferriprox®) (*^1^) or deferoxamine (Desferal®) (*^2^); *^3^documented incompliance. Shadowed patients undergoing 2nd SCT.

Cross-match analyses to detect donor-specific antibodies (DSA) were performed in all patients, using the Single Antigen Bead (SAB) test from Thermo Fisher Scientific (Onelambda). Donor selection hierarchy prioritized the absence of DSA followed by major blood group incompatibility and cytomegaly virus (CMV) serology match. In case of only one available haploidentical donor, an autologous bone marrow (BM) backup was collected for rescue in case of a GF.

Two patients received a T-haplo-HSCT as a second transplant. One patient endured a GF six months after a MUD transplantation; one a prior maternal haplo-HSCT with secondary GF, 1,5 months post-HSCT.

### Ethics approval and consent to participate

Written and informed consent was obtained from all patients and/or their parents/legal guardians in accordance with the Declaration of Helsinki. All methods were performed in accordance with local regulations applicable at the time of transplantation. The trial was reviewed and approved by the Institutional Review Board of the University of Regensburg (Reg.-No. 25-4099-104). Due to the retrospective nature of this study and the de-identification of participants, informed consent was deemed unnecessary.

### Donors and grafts

T-haplo-HSCT grafts were of parental origin (median age: 38 years; range 28-50), sharing one HLA haplotype (Table 1). Patients with a MSD (median age: 15 years; range: 12-25 years) received a BM allograft. In T-haplo-HSCT, peripheral blood harvests were depleted of CD3^+^ or αβ T-cells, and CD19^+^ B cells using the CliniMACS device. Targeted cell counts were CD34^+^ >1 ×10^7^/kg body weight (BW), CD3^+^/TCRαβ^+^ <1 ×10^5^/kg BW, and CD19^+^ <5 ×10^5^/kg BW (Table 2). Thirteen patients received 14 haplo-HSCT (three CD3/CD19-depleted and eleven TCRab/CD19-depleted cell products); seven received a MSD HSCT. The method of T cell depletion was changed during the course of the study as the refined alpha/beta T cell depletion became more readily available.

**Table 2:** Graft contents and engraftment characteristics.

Graft content (median)	MSD	T-haplo-HSCT	TCRαβ/CD19	CD3/CD19
CD34^+^ cells/kg BW		15 (9–24) × 10^6^	17 (10–24) ×10^6^	12 (9–22) ×10^6^
TNC/kg BW	5 (3–8) x 10^8^			
CD3^+^ T cells/kg BW			571 (140–2915) ×10^4^	10 (0.7–42) ×10^4^
TCRαβ/ CD3+ T cells/ kg BW			0.4 (0.1–1.6) ×10^4^	
CD19^+^ B cells/kg BW		4 (0.4–12) ×10^4^	4 (2–11) ×10^4^	3 (0.4–12) ×10^4^
Time to Engraftment (days; median)				
Leukocyte >1 ×10^9 ^L^–1^	29 (20–37)	13 (11–36)	14 (11–36)	12 (11–18)
Granulocyte >0.5 ×10^9 ^L^–1^	31 (20–43)	17 (11–35)	17 (11–35)	16 (13–18)
Thrombocyte >20 ×10^9 ^L^–1^	40 (25–406)	15 (12–36)	15 (12–36)	16 (15–17)

Absolute cell doses per kilogram (kg) body weight (BW) and days of engraftment values are shown as median (minimum-maximum values).

*T-haplo-HSCT* indicates patients who underwent T-haplo-HSCT (both T-cell depletion procedures), *CD3/CD19* patients only with CD3/CD19 T-cell depletion, *TCRαβ/CD19* patients only with TCRαβ/CD19 T-cell depletion, *MSD* patients with an HLA identical donor (matched sibling donor).

### Conditioning regimen and immune therapy

No hypertransfusion/pretransplant pharmacologic immunosuppression (PTIS) was applied. The conditioning regimen consisted of thiotepa (5 mg/kg; day -8 and -7), fludarabine (40 mg/m^2^; day −7 to −4), and treosulfan (14 g/m^2^; day −4 to −2 (haplo-HSCT) (FTT), respectively. In the haplo-HSCT group, one patient with allo- and autoimmune hemolytic anemia received bortezomib and rituximab prior to transplant and additionally received total body irradiation (TBI) (2 Gy) during the conditioning regimen. Pre-transplantation anti-thymocyte globulin (ATG Grafalon®, Neovii, Gräfelfing, Germany) was administered from day −3 to −1 at 10 mg/kg/day for GvHD prophylaxis in MSD and day −10 to −8 at 15 mg/kg/day in T-haplo-HSCT for prevention of graft rejection. Post-transplant immunosuppression consisted of tacrolimus (FK506; target level: 5–8 ng/ml) as a 20h-continous infusion in addition to mycophenolate mofetil (MMF) 600 mg/m^2^ starting day -3 and day −1, respectively. Cyclosporine A (CsA) (target level: 120–150 ng/mL), initially used in four patients (1 MSD, 3 haplo-HSCT), was replaced by tacrolimus as continious20 hours infusion to prevent neurological complications such as posterior reversible encephalopathy syndrome (PRES). Immunosuppression was to be continued until day +240 in the haplo-HSCT and day +180 in the MSD cohort, respectively. Weaning of immunosuppression was adjusted depending on chimerism and viral reactivation. In case of falling donor cell chimerism during the immunosuppressant withdrawal process, split-chimerism analysis were performed. With a donor derived myeloid CD14 split-chimerism above 90% immunosuppression was continued until donor CD3^+^ chimerism reached 50%. In case of viral reactivation, and an almost complete donor derived CD14 split-chimerism, a decreased donor derived CD3 split-chimerism was interpreted as reactivation of autologous virus-specific T cells and immunosuppression was reduced and if necessary discontinued earlier.

### Chimerism analysis and immunologic reconstitution

Peripheral blood (PB) chimerism was performed via variable number of tandem repeat (VNTR) polymorphisms beginning with engraftment and at least until day +180, and monthly thereafter in case of stable chimerism. In case of a donor derived mixed chimerism (MC; <90%), split-chimerism analyses were performed on mononuclear cell (MNC) fractions, sorted on a BD FACSAria™ cell sorter (Becton Dickinson), both from PB and BM [26]. MNC were sorted using anti-CD3-FITC (BD until 2020; Biolegend since 2021), anti-CD14-PE ((Miltenyi Biotec), anti-CD19-APC (eBioscience until 2020; Biolegend since 2021) and anti-CD45-PerCP (Miltenyi Biotec) and anti-CD45 BUV395 (BD; since 2021). Erythroid precursors were labeled withCD235a-APC (Miltenyi Biotec) and sorted as CD45^-^CD235a^+^ from BM cells.

GF was defined as an absence of donor engraftment, autologous hematopoietic reconstitution, or receiving a second transplantation, where primary GF was defined as persistence of marrow aplasia at day 28 post-HSCT and secondary GF as loss of donor cells after transient engraftment with subsequent return to transfusion dependence.

Neutrophil engraftment was defined as the first of three consecutive days of a neutrophil count greater than 0,5 ×10^9^/L; platelet recovery was defined as an unsupported platelet count greater than 20 ×10^9^/L for seven consecutive days. T-cell recovery was defined as T-cell counts >200/µl. Transfusion independence was defined as a weighted average hemoglobin level of at least 9 g per deciliter without transfusion for at least six consecutive months throughout the study period.

### Supportive care and monitoring

Supportive care was performed according to institutional standards. In case of CMV disparity, ganciclovir (until 2017) or letermovir (since 2018) [27] was administered as antiviral prophylaxis. Weekly PCR-monitoring for EBV, CMV, human herpes virus 6 (HHV6), adenovirus (ADV), and BK-polyomavirus (BKV) was routinely performed until withdrawal of immunosuppression. Viral reactivation was defined as a copy number >10^3^ copies/mL or the start of therapeutic measures. Viral disease was defined as the presence of clinical symptoms in addition to viral reactivation.

### Statistical analysis

Rejection, recurrence of TDT, and death were recorded as events. DFS was defined as transfusion independence. Transplantation-related mortality (TRM) was defined as all causes of death related to the transplantation procedure.

## Results

### Overall survival, event-free and disease-free survival

The patient and donor characteristics are summarized in Table 1. In haplo-HSCT patients with a median follow-up of 39.5 months (range: 6.5–90.6 months), the OS was 100% and EFS and DFS were 92%, respectively, whereas OS, EFS and DFS were 100% in MSD patients with a median follow-up of 35 months (range: 20–74 months). No transplant-related mortality was observed in either patient cohort. Hospitalization in the haplo-HSCT cohort was a median of 45 days (range 25–86 days), and 53 days (range 46–58 days) in the MSD cohort, respectively.

### Engraftment, graft failure/rejection and immunological reconstitution

All but one patient experienced primary engraftment. One patient who received a haplo-HSCT with CD3^+^/CD19^+^ T-cell depletion experienced early GF and underwent a second successful haplo-HSCT by an alternative haploidentical donor. One patient who received a haplo-HSCT with TCR αβ/CD19^+^ depletion developed a late GF 11 months after initial HSCT and 2 months after weaning of immunosuppression, despite receiving two stem cell boosts and is currently awaiting a potential second HSCT. Median chimerism (PB) was 100% (range: 84–100%) in the haplo-HSCT group versus 92% (range: 27.5–100%) in the MSD group at last follow-up. Complete and sustained engraftment of donor origin (complete donor derived chimerism >90%) was documented in 4/7 (57%) of patients undergoing MSD HSCT compared to 9/13 (69%) of haplo-HSCT patients, with one patient achieving sustained engraftment after undergoing a second haplo-HSCT following primary GF. Mixed donor derived chimerism <90% (MC) was observed in 3/7 (43%) MSD-HSCT patients vs. 3/13 (23%) haplo-HSCT patients, with all patients remaining transfusion-independent. The split-chimerism analyses of the T- haplo-HSCT patients showed a nearly complete myeloid chimerism and a stable mixed chimerism in the CD3 and CD19 fraction after weaning of immunosuppression. The three MSD patients presented with a myeloid chimerism of 16.2%, 67.2% and 100% after weaning of immunosuppression. Interestingly, the MSD patient with a myeloid chimerism of 16.2% (total 27.7%) at last follow-up maintains a sustained graft function and is transfusion independent with a stable hemoglobin level around 11.5–12 g/dl. The stable mixed total chimerism post-immunosuppression, mostly due to the autologous lymphocyte fraction, was noticed. Due to immune tolerance graft maintenance was not jeopardize.

**Fig. 1:**
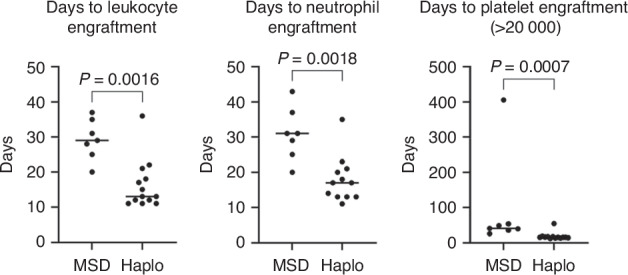
Time to engraftment. The time to engraftment for leukocytes (>1 ×10^9^/L), neutrophiles (>0.5 ×10^9^/L) and platelets >20 ×10^9^/L) was significantly shorter for patients receiving a T-Haplo HSCT vs. a MSD HSCT. Data was entered into GraphPad Prism Version 10.2.3 for statistical analysis and graphed as a scatter dot plot. Individual values are displayed, with the line representing the median time to reach engraftment. Statistical analysis was done by first accessing normality visually and via the Shapiro–Wilk test, followed by an unpaired nonparametric Mann–Whitney test with a confidence interval of 95% and calculation of a two-tailed *p*-value. Definition of statistical significance *P* < 0.05.

Median recovery time in haplo-HSCT and MSD was significantly different with 13 (11–36) and 29 (20–37) days for leukocytes (>1 ×10^9^/L), 17 (range 11–35) and 31 (range 20–43) days for neutrophils (>0.5 ×10^9^/L), and 15 (range 12–36) and 40 (range 25–406) days post-HSCT for thrombocytes (>20 ×10^9^/L), respectively (Table 2 and Fig. 1). All three patients who underwent a second stem cell transplantation (one due to early GF transplanted at our center and two due to earlier failed HSCT attempts) in the T-haplo-HSCT group experienced successful long-term engraftment. The median hemoglobin level raised from 7.6 g/dl (range: 6.8–8.8) pre- to 11.8 g/dl (range: 8.5–14.2) post-HSCT in MSD and from 7.9 g/dl (range: 7.2–8.9) to 10.8 g/dl (range: 8.8–13) in haplo-HSCT. Transfusion independence was achieved on day 34 (range: 8–95) and 84 (range: 6–257) in MSD and T-haplo-HSCT, respectively, and on day 39 (range 6–224) in the TCR αβ/CD19^+^ subset.

**Fig. 2:**
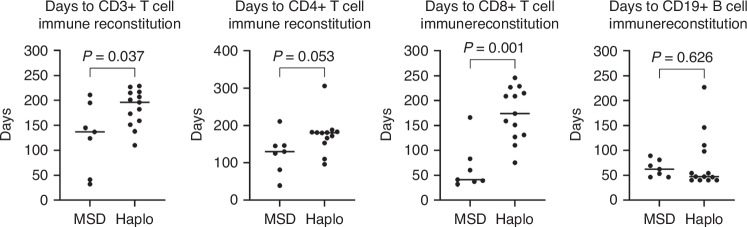
Time to immune reconstitution. Data was entered into GraphPad Prism Version 10.2.3 for statistical analysis and graphed as a scatter dot plot. Individual values are displayed, with the line representing the median time to reach engraftment. Statistical analysis was done by first accessing normality visually and via the Shapiro–Wilk test, followed by an unpaired nonparametric Mann–Whitney test with a confidence interval of 95% and calculation of a two-tailed *p*-value. Definition of statistical significance *P* < 0.05.

Immunosuppression was terminated after a median of 220 (range: 88–527) and 178 (range: 108–296) days in haplo-HSCT and MSD patients, respectively. The median time to reach >0.5 ×10^9^/L CD3^+^ T cells in the T-haplo-HSCT group compared to the MSD group was 192 (110–229) and 137 (32–211) days, respectively. CD4^+^ T cells (>0.2 ×10^9^/L) recovered by days 180 (96–306) and 130 (39–211) in haplo-HSCT and MSD patients, respectively. B-cell recovery (>0.1 ×10^9^ L-1) was reached by day 47 (range: 40–224) and 62 (range: 46–89) for haplo-HSCT and MSD patients, respectively (Table 3 and Fig. 2). Immune reconstitution was significantly lower in the MSD group regarding CD3+ T cells and CD8+ T cells (Fig. 2).

**Table 3:** Immune reconstitution of patients after SCT for β-Thalassemia.

T-and B cell recovery	MSD	T-haplo-HSCT	TCRα/β/CD19	CD3/CD19
CD3^+^ T cells > 0.5 ×10^9 ^L^−1^	137 (32–211)	192 (110–229)	201 (138–229)	173 (110–227)
CD4^+^ T cells > 0.2 ×10^9 ^L^−1^	130 (39–211)	180 (96–306)^a^	176 (96–188)^a^	181 (110–306)
CD8^+^ T cells > 0.2 ×10^9 ^L^−1^	41 (32–166)	167 (75–246)	174 (127–246)	110 (75–227)
CD19^+^ B cells > 0.1 ×10^9 ^L^−1^	62 (46–89)	47 (40–224)	47 (40–146)	110 (40–227)

Median (minimum-maximum) values are shown as days after SCT and percentages, respectively; *T-haplo-HSCT* indicates patients who underwent T-haplo-HSCT (both T-cell depletion procedures), *CD3/CD19* patients only with CD3/CD19 T-cell depletion, *TCRαβ/CD19* patients only with TCRαβ/CD19 T-cell depletion, *MSD* patients with HLA identical donor (matched sibling donor).

^a^One value missing as patient has not yet reached necessary cell count.

### Regimen-related toxicities, and infectious complications

The treosulfan-based conditioning regimen was well tolerated. In particular, no VOD/SOS was observed in this high-risk population. Most early complications were headache, grade I-II mucositis, fever of unknown origin, diarrhea, and episodes of pain in bone and muscles, mostly associated with the time immediately before and during the early engraftment period. Four patients experienced transient grade III or IV toxicity: in the T-haplo-HSCT group, one patient experienced a transfusion-related acute lung injury (TRALI) within one week after HSCT and another developed grade IV mucositis after conditioning. In the MSD group, one patient was diagnosed with an acute respiratory distress syndrome (ARDS)/capillary leak syndrome and mucositis grade IV during/after conditioning. All toxicities resolved. Relevant neurotoxicity (NT), particularly PRES, was seen in only one patient receiving CsA (T-haplo-HSCT group). After switching immunosuppression to everolimus, the symptoms resolved. Another patient developed transient idiopathic Bell’s Palsy.

Three patients developed renal complications. In the MSD cohort, one patient developed mild Fanconi Tubulopathy with no further therapeutic intervention. Within the T-haplo-HSCT cohort, two patients developed renal insufficiency; one patient suffered from acute renal insufficiency grade I, following conditioning chemotherapy, which has resolved; a second patient developed mild stable chronic renal insufficiency, with no further for therapeutic intervention. Three patients (T-haplo-HSCT group) developed endocrinologic disorders with onset post-HSCT. In the CD3/CD19 subset, one patient presented with growth retardation while another suffered from hypogonadism and hypothyroidism following TBI treated with hormone replacement therapy, as well as CsA-associated osteoporosis. In the TCR αβ/CD19 subset, one patient developed autoimmune thyroiditis following HSCT, treated with hormone substitution.

Four patients (one MSD, three T-haplo-HSCT cohort) developed transient mild autoimmune disorders (hemolytic anemia, *n* = 2; immune thrombocytopenia *n* = 2). One patient has been treated with erythropoietin and one with eltrombopag and romiplostim. All four patients are medication- and transfusion-independent at latest follow-up. Two patients (T-haplo-HSCT cohort) presented with hypogammaglobulinemia following Rituximab therapy and received IVIG.

No grade IV bacterial or fungal infection was observed. In the MSD population, two patients (29%) developed viral disease (Table 4): one experienced ADV-positive diarrhea, another developed transient dysuria and microhematuria with BKV reactivation. Both resolved with symptomatic treatment only. In the haplo-HSCT population, six patients (30%) developed viral disease (Table 4) with three patients (23%) receiving additionally virus-specific T-cells. All viral diseases resolved.

**Table 4:** Incidence of infections in patients after SCT for β-thalassemia.

	All	MSD	T-haplo-HSCT	TCR αβ/CD19	CD3/CD19
Bacteremia/sepsis (isolated pathogen)	6/20 (30%)	3/7 (43%)	3/13 (23%)	3/10 (30%)	0/3 (0%)
Viral reactivation and/or disease	20/20 (100%)	7/7 (100%)	13/13 (100%)	10/10 (100%)	3/3 (100%)
Virus-specific pharmaceutical treatment	9/20 (45%)	0/7 (0%)	9/13 (69%)	7/10 (70%)	2/3 (66%)
Cellular treatment (VSTC)	3/20 (15%)	0/7 (0%)	3/13 (23%)	2/10 (20%)	1/3 (33%)
Viral reactivation (any)	20/20 (100%)	7/7 (100%)	13/13 (100%)	10/10 (100%)	3/3 (100%)
- CMV (blood)	8/20 (40%)	2/7 (29%)	6/13 (46%)	4/10 (40%)	2/3 (66%)
- HHV6 (blood)	8/20 (40%)	1/7 (14%)	7/13 (54%)	4/10 (40%)	3/3 (100%)
- EBV (blood)	7/20 (35%)	2/7 (29%)	5/13 (38%)	2/10 (20%)	3/3 (100%)
- BKV (blood)	7/20 (35%)	3/7 (43%)	4/13 (31%)	3/10 (30%)	1/3 (33%)
- ADV (blood)	3/20 (15%)	0/7 (0%)	3/13 (23%)	2/10 (20%)	1/3 (33%)
- CMV (urine)	6/20 (30%)	0/7 (0%)	6/13 (46%)	5/10 (50%)	1/3 (33%)
- BKV (urine)	9/20 (45%)	3/7 (43%)	6/13 (46%)	4/10 (40%)	2/3 (66%)
- ADV (stool)	11/20 (55%)	6/7 (86%)	7/13 (54%)	5/10 (50%)	2/3 (66%)
Viral Disease (any)	9/20 (45%)	2/7 (29%)	7/13 (54%)	6/10 (60%)	1/3 (33%)
- CMV (blood)	2/20 (10%)	0/7 (0%)	2/13 (15%)	1/10 (10%)	1/3 (33%)
- EBV (blood)	1/20 (5%)	0/7 (0%)	1/13 (8%)	1/10 (10%)	0/3 (0%)
- BKV cystitis	2/20 (10%)	1/7 (14%)	1/13 (8%)	1/10 (10%)	0/3 (0%)
- ADV (stool)	4/20 (20%)	1/7 (14%)	3/13 (23%)	3/10 (30%)	0/3 (0%)

*Viral reactivation* is defined as copy numbers >1000/mL or copy numbers <1000/mL +treatment, *viral disease* is defined as viral reactivation +symptoms, *T-haplo-HSCT* indicates patients who underwent T-haplo-SCT (both T-cell depletion procedures), *CD3/CD19* patients only with CD3/CD19 T-cell depletion, *TCR αβ/CD19* patients only with T-cell depletion, *MSD* patients with HLA identical donor (matched sibling donor), *ADV* adenovirus, *BKV* polyomavirus, *CMV* cytomegalovirus, *EBV* Epstein-Barr virus, *HHV6* human herpesvirus 6, *HSV* herpes simplex virus, *VSTC* virus-specific T-cell, *VZV* varicella zoster virus.

In case of CMV disparity, ganciclovir (until 2017) or letermovir (since 2018) were administered as antiviral prophylaxis with 66,6% of patients without CMV prophylaxis versus 36,3% of patients with letermovir prophylaxis experiencing CMV reactivation. Neither of the two patients with CMV-associated viral disease was on letermovir prophylaxis.

### Graft-versus-host disease (GvHD)

No MSD patient experienced aGvHD or cGvHD. Of the T-haplo-HSCT cohort, five patients (38%) experienced mild aGvHD grade I-II, which resolved with prednisolone, topical tacrolimus and extracorporal photopheresis (ECP). None of the patients developed aGvHD grade III or IV. Two patients in the T-haplo-HSCT cohort developed mild to moderate cGvHD. One of the patients with aGvHD also developed mild cGvHD of the skin, which also resolved with prednisolone and ECP. Another adult patient in the T-haplo-HSCT group developed a moderate steroid-sensitive cGvHD with cutaneous, oral, and hepatic involvement. cGvHD completely resolved under prolonged immunosuppression with MMF, everolimus and prednisolone.

## Discussion

Thirteen patients with TDT with severe iron overload received a CD3^+^- or aß-depleted haplo-HSCT and were compared to seven patients who received a MSD transplantation using an almost identical treosulfan-based conditioning regimen. HSCT engraftment and transfusion independence with T-haplo-HSCT were achieved in all but one very early post-transplant with normalized total hemoglobin levels and remained stable throughout the follow-up. Mean engraftment of neutrophils and platelets, as well as transfusion-independence, were comparable to the findings of several other study groups investigating T-haplo-HSCT [13, 16, 20, 21]. The OS of the haploidentical group, although still small, is 100%, compared to the outcome in the European Society for Blood and Marrow Transplantation (EBMT) registry, where the reported 2-yrs OS in MD HSCT is 92.1% in children and only 84.4% in adults [4]. EFS is reportedly age- and donor-dependent with 80% and 63% in patients 7 to 15 years and 16 to 25 years, respectively, and 86% and 70% for MSD and haplo-HSCT, respectively [28]. Accordingly, this series presented with a superior EFS.

Since patients with a hypertransfusion and an otherwise intact immune system are at substantial risk for rejection/GF, PTIS with dexamethasone and fludarabine are used in haplo-HSCT using post-CY [20]. PTIS was not applied in our setting, yet we did not observe an acute graft rejection in this patient cohort. This is in line with the experience in our series of patients transplanted for SCD [24]. The prolonged post-transplant immunosuppression of approximately 6 months with MMF and tacrolimus and a chimerism-triggered weaning seems to protect donor T cells, consecutively allowing a stable engraftment with an almost full donor chimerism off immunosuppression. In mixed chimera, split-chimerism analyses showed a dissociated split-chimerism with a full myeloid chimerism and transfusion-independence. We therefore consider split-chimerism analyses obligatory in all patients who develop a mixed chimerism before any action is taken.

FTT is emerging as the standard of care myeloablative regimen for hemoglobinopathies. The observed benign adverse event profile of treosulfan in TDT is in line with recent publications and is significantly superior to busulfan-based regimens [29].

It is particularly noteworthy that no VOD/SOS was observed, as the reported incidence of VOD/SOS in TDT is currently on average 10% [14]. This can be exceeded with additional risk factors such as busulfan, younger age, and severe hepatic iron overload. In class III patients conditioned with busulfan, the incidence was 78% [14]. When Busulfan is used as single agent conditioning in the gene therapy/editing trials for TDT, the reported incidences range from 10% to 17.4% [15]. Interestingly, this risk is considered significant by the FDA to recommend VOD/SOS prophylaxis (https://www.fda.gov/media/174610 and https://www.fda.gov/media/174615/download?attachment).

Viral reactivation and disease are one of the expected complications of a T-haplo-HSCT. Albeit viral reactivation was observed, no persistent viral disease emerged, despite prolonged immunosuppression. CMV reactivation, a frequent serious complication, was efficiently controlled with letermovir prophylaxis, similar to our SCD population with significantly reduced TRM after the introduction of this measure [30].

Acute and chronic GvHD is next to DFS the most pivotal outcome parameter in non-malignant disease. Neither grade III-IV acute nor severe chronic GvHD was observed and is in line with other series in non-malignant diseases [9, 24].

In summary, aß T cell-depleted haplo-HSCT is safe and effective in TDT, with outcomes comparable to MSD transplantation. Readily available donors, a low risk of TRM and GvHD, and a low risk of VOD/SOS in this high-risk patient population render FTT-based T-haplo-HSCT a safe and efficient approach for TDT patients. One limitation of the study was the relatively small number of patients included. However, this treatment concept is being already investigated in a prospective international study (EudraCT No. 2018-002652-33) in patients with SCD. This proof-of-efficacy in patients with TDT now allows this prospective evaluation to be expanded.

## Data Availability

All data supporting the findings of this study are available within the paper and its Supplementary Information. Additional sensitive data are available from the corresponding author upon reasonable request.
